# Model-averaged Bayesian *t* tests

**DOI:** 10.3758/s13423-024-02590-5

**Published:** 2024-11-07

**Authors:** Maximilian Maier, František Bartoš, Daniel S. Quintana, Fabian Dablander, Don van den Bergh, Maarten Marsman, Alexander Ly, Eric-Jan Wagenmakers

**Affiliations:** 1 Department of Experimental Psychology, University College London, 26 Bedford Way 129-B, WC1H 0AP London, UK; 2 Department of Psychology, University of Amsterdam, Amsterdam, The Netherlands; 3 Institute of Computer Science, Czech Academy of Sciences, Prague, Czechia; 4 Department of Psychology, University of Oslo, Oslo, Norway; 5 NevSom, Department of Rare Disorders, Oslo University Hospital, Oslo, Norway; 6 KG Jebsen Centre for Neurodevelopmental Disorders, University of Oslo, Oslo, Norway; 7 Institute for Advanced Study, University of Amsterdam, Amsterdam, Netherlands; 8 Institute for Biodiversity and Ecosystem Dynamics, University of Amsterdam, Amsterdam, Netherlands; 9 Machine Learning Group, CWI Amsterdam, Amsterdam, The Netherlands

**Keywords:** Bayesian model-averaging, *t* test, Bayes factor, *t*-likelihood, Robust inference, Unequal variances

## Abstract

One of the most common statistical analyses in experimental psychology concerns the comparison of two means using the frequentist *t* test. However, frequentist *t* tests do not quantify evidence and require various assumption tests. Recently, popularized Bayesian *t* tests do quantify evidence, but these were developed for scenarios where the two populations are assumed to have the same variance. As an alternative to both methods, we outline a comprehensive *t* test framework based on Bayesian model averaging. This new *t* test framework simultaneously takes into account models that assume equal and unequal variances, and models that use *t*-likelihoods to improve robustness to outliers. The resulting inference is based on a weighted average across the entire model ensemble, with higher weights assigned to models that predicted the observed data well. This new *t* test framework provides an integrated approach to assumption checks and inference by applying a series of pertinent models to the data simultaneously rather than sequentially. The integrated Bayesian model-averaged *t* tests achieve robustness without having to commit to a single model following a series of assumption checks. To facilitate practical applications, we provide user-friendly implementations in JASP and via the $\texttt {RoBTT}$ package in $\texttt {R}$. A tutorial video is available at https://www.youtube.com/watch?v=EcuzGTIcorQ

The independent-samples *t* test assesses the difference between two group means; consequently, it is one of the most common analytical techniques in experimental psychology. Indeed, Wetzels et al. (2011) show that psychologists report on average 3.4 *t* tests per article, resulting in one *t* test for every 2.8 pages. In conversation, one of our former colleagues has even expressed the opinion that “all interesting scientific questions in psychology can be broken down into two groups and a *t* test”. These *t* tests are almost always conducted within the framework of frequentist statistics, with a *p* value as the final metric of interest. However, in recent years, several Bayesian *t* tests have been proposed to assess the difference in group means using Bayes factors (Gönen et al., 2005; Rouder et al., 2009; Gronau et al., 2020). These Bayes factor tests offer several advantages over the frequentist *t* tests based on *p* values. First, the Bayesian framework does not force the researcher into making an all-or-none decision to reject or accept a hypothesis, since Bayes factors provide a continuous measure of the strength of evidence (Wagenmakers et al., 2016). Second, the Bayesian framework generalizes seamlessly to sequential analysis. Unlike *p* values, Bayes factors are *consistent* under both the null and the alternative hypothesis, meaning that as data accumulate indefinitely, the chance that the Bayes factor points to the correct hypothesis approaches 1. This property enables hypothesis testers to stop whenever the evidence is deemed to be sufficiently compelling, and this allows for a flexible testing regime that is both efficient and ethical (Berger and Wolpert, 1988; Edwards et al., 1963; Rouder, 2014; Schönbrodt et al., 2017; Stefan et al., 2020; Wagenmakers et al., 2022; for a discussion, see de Heide and Grünwald, 2021; Hendriksen et al., 2021; Sanborn and Hills, 2014).^1^ Third, the Bayesian framework allows researchers to quantify evidence in favor of the null hypothesis as well as the alternative hypothesis (Gallistel, 2009; Rouder et al., 2009). This allows a key distinction to be made between “absence of evidence” and “evidence of absence” (Keysers et al., 2020).

The currently popular Bayesian *t* tests are limited to the equal variance case (Gönen et al., 2005; Morey & Rouder, 2015; Rouder et al., 2009). Bayesian *t* tests for unequal variances were already pioneered by Dickey in the 1970s (Dayal & Dickey, 1976; Dickey, 1976, 1973, 1977), with other versions of unequal-variance *t* tests proposed later (Barbieri et al., 2016; Bartolucci et al., 1998; Fu et al., 2020; Moreno et al., 1999; Wetzels et al., 2009). Currently, however, no unequal-variance Bayesian *t* test is readily available in popular statistical software packages and no method allows researchers to define priors on an intuitive scale or in a way that is appropriate for testing (i.e., using a prior that is not data dependent). This is surprising given that unequal variances are common in psychology and the unequal variance scenario is more flexible (Delacre et al., 2017; Erceg-Hurn & Mirosevich, 2008; Grissom, 2000; Keselman et al., 1998).

A related problem is that in practice, it can be difficult to determine whether or not the variances are equal, especially when sample sizes are small. In the frequentist framework, inference usually follows a two-step approach: the first step is to test for unequal variances, and the second step, contingent on the outcome of the first, is to conduct either the Student’s *t* test or the Welch test (for a summary of examples see Hayes & Cai, 2007, p. 219).^2^ However, this two-step approach fails to take into account the uncertainty about unequal variances; moreover, it tempts researchers into assuming the variances are equal even though the power to detect unequal variances may be low.

In the Bayesian framework, the conundrum of what model to select can be avoided using model-averaging or multi-model inference, a technique that takes into account all pertinent models simultaneously, weighting their impact with respect to their predictive performance (Hinne et al., 2020; Hoeting et al., 1999). Specifically, in Bayesian model-averaging, parameters are estimated for all models under consideration (i.e., both equal-variance and unequal-variance models) and these estimates are then averaged based on how well the associated models predicted the observed data. Consequently, with Bayesian model-averaging researchers can draw inferences from equal-variance and unequal-variance models simultaneously, where each model’s relative inferential impact is determined by its relative plausibility. This way model-averaging circumvents the problem associated with sequentially performing all-or-none decisions.

An additional concern with standard *t* tests is their sensitivity to outliers; a few extreme observations can exert a large impact on the value of the *t*-statistic and thereby have a disproportional effect on the resulting test. Several methods may be used to mitigate the impact of outliers (e.g., Mair and Wilcox, 2020; Wilcox, 2017), and here we focus on a relatively straightforward solution, namely to consider models where the *t*-likelihood is used in place of the normal likelihood. A *t*-distribution has fatter tails, which means that relatively extreme observations have a higher chance of occurring. The use of a *t*-likelihood is not new, but previous work typically focused on parameter estimation without simultaneously taking the normal likelihood under consideration (e.g., Bayarri and Mayoral, 2002; Gelman and Hill, 2006, p. 124; Kruschke, 2013; Kruschke, 2018; O’Hagan and Forster, 2004, pp. 223-231; Western, 1995)

In practice, it can be difficult to ascertain whether or not outliers are present, and whether or not their presence warrants the application of a robust method. Consequently, researchers usually apply intuitive but potentially problematic decision strategies, such as “histomancy” – the attempt to derive likelihood functions from gazing at empirical histograms (McElreath, 2016, p. 326). Bayesian model-averaging resolves the tension between robust and classical methods because the data determine the degree to which inference is based on robust models versus standard models. Importantly, the extent of this difference is gradual instead of all-or-none.

The goals of this manuscript are threefold. First, we present an unequal-variance *t* test (i.e., a Bayesian Welch *t* test) which requires that prior distributions are assigned to Cohen’s $\delta$^3^ and to the relative size of the precision. Second, we combine the unequal-variance *t* test with the equal-variance *t* test (i.e., the Bayesian Student’s *t* test) into a model-averaged *t* test (i.e., a model-averaged Bayesian *t* test, MB *t* test). Third, we extend the model-averaged *t* test by adding models with a *t*-likelihood to the model ensemble (i.e., a robust model-averaged Bayesian *t* test, RoMB *t* test). By averaging across the entire ensemble of eight models, robust answers can be obtained to the following questions:

1. What is the evidence for the presence vs. the absence of a difference in means?
2. What is the evidence for equal vs. unequal variances?
3. What is the evidence for normal likelihoods vs. *t*-likelihoods?

We illustrate the new methodology with the data of Pleasant and Barclay (2018) who investigated why highly cooperative people sometimes get punished (“antisocial punishment”) and with the data of Roozenbeek et al. (2021) who showed that an accuracy nudge intervention can increase the discernment between true and fake news on social media. To facilitate application in practice we also implemented the *t* tests in the RoBTT R package (Bartoš & Maier, 2022).

## Bayesian Student’s *t* test

Before proceeding to the case of unequal variances, we briefly revisit the Bayesian equal-variance *t* test (i.e., Rouder et al., 2009).^4^ The Bayesian Student’s *t* test contrasts two competing hypotheses: The null hypothesis $\mathcal {H}_0$, which assumes that the data are normally distributed, that the variances are equal between the groups, and that the effect of the intervention is absent (i.e., the true difference in means between the intervention and the control group is zero), and the alternative hypothesis $\mathcal {H}_1$, which additionally assumes that there exists a true non-zero difference in means between the two groups. The corresponding models of the data-generating process can be found in Appendix B. The evidence for either hypothesis is quantified using the Bayes factor (see InfoBox 1), which compares the marginal likelihood of the data under the alternative hypothesis to the marginal likelihood of the data under the null hypothesis.^5^

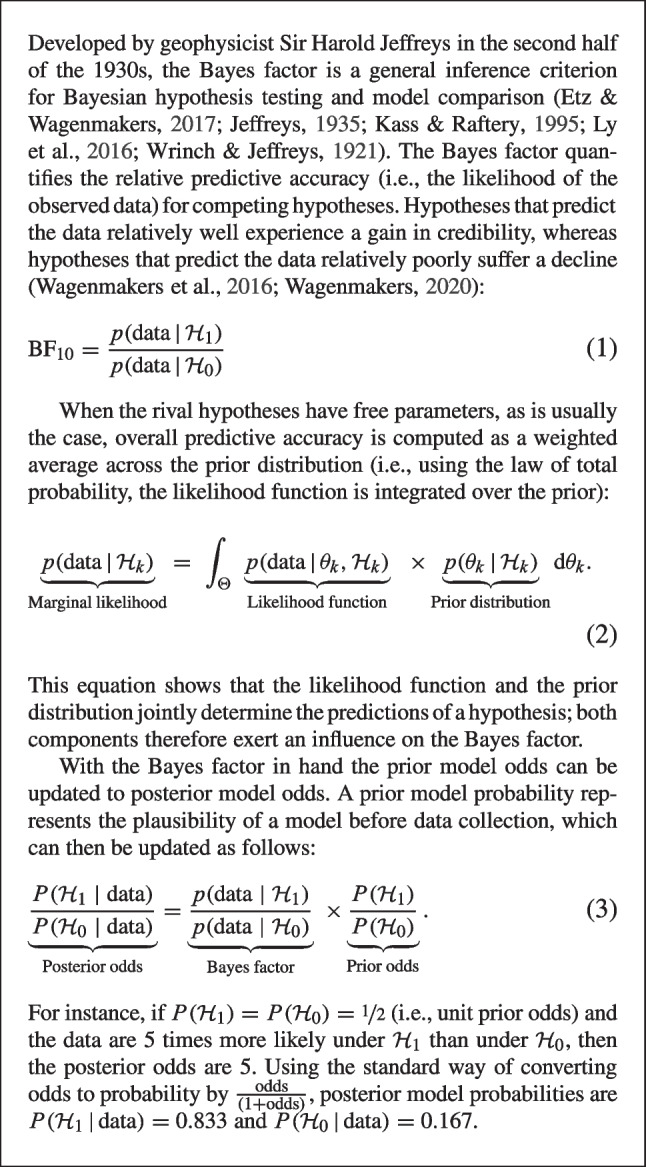



Although Bayes factors (BF) are a continuous measure of the strength of evidence and any discretization will inevitably result in loss of information, the following rule of thumb may help with interpretation: $1< \text {BF} < 3$ corresponds to weak evidence, $3< \text {BF} < 10$ corresponds to moderate evidence, and $\text {BF} > 10$ corresponds to strong evidence (e.g., Jeffreys, 1939; Lee and Wagenmakers, 2013, p. 105; Wasserman, 2000). When considering the evidence for the null rather than the alternative, the Bayes factor can simply be inverted (i.e., $\text {BF}_{01} = \nicefrac {1}{\text {BF}_{10}}$).

### Prior distributions

A unique part of any Bayesian analysis is the appropriate specification of parameter prior distributions (e.g., Stefan et al., 2020). The crucial prior distribution for the equal-variance *t* test is the prior on the standardized difference between the group means, that is, on Cohen’s $\delta$, the population version of Cohen’s *d*. We follow Rouder et al. (2009) and assign $\delta$ a Cauchy(0, 1/$\sqrt{2}$) distribution.^6^

However, the researcher is free to adopt their own prior distribution, tailored to their specific research question. For example, when the direction of the effect and its likely size are known it can be more efficient to adopt an informed prior (e.g., Gronau et al., 2017; Vohs et al., 2021); similarly, one may adopt prior distributions that are informed by past data (e.g., Bartoš et al., 2021; Ibrahim et al., 2015).

In the case of two models, the Bayes factor quantifies the evidence provided by the data, independent of the prior plausibility of the models (cf. Eq. 1). Throughout this work we adhere to Jeffreys’s simplicity postulate and take on a position of equipoise: $p(\mathcal {H}_0) = p(\mathcal {H}_1) = \nicefrac {1}{2}$ (e.g., Jeffreys, 1950, p. 316). This means that the relative plausibility of the competing models is determined solely by their relative predictive performance for the observed data.

### Running example

**Fig. 1:**
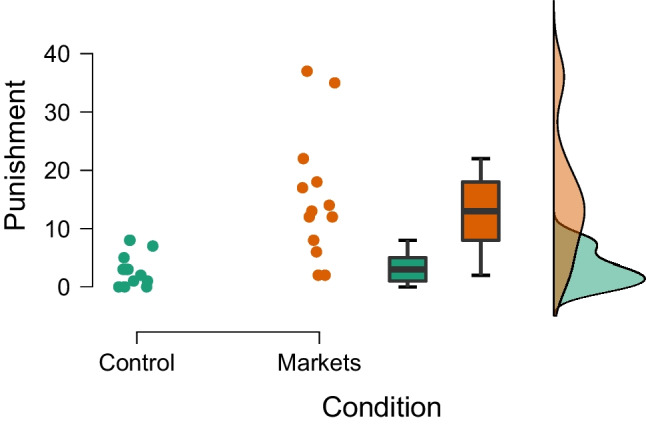
Social punishment in the control condition vs. the biological markets condition. Data from Pleasant and Barclay (2018), available at https://tinyurl.com/mwpuhpx8. Figure created in JASP: JASP Team, (2022)

Let us illustrate the Bayesian equal-variance *t* test on the data of Pleasant and Barclay (2018), who investigated why people sometimes show “antisocial punishment” (Fig. 1). Pleasant and Barclay (2018) predicted that antisocial punishment would occur more often in a “biological markets condition” (i.e., where participants compete to play a trust game with a third-party individual) than in a control condition. The third-party individual saw the average contributions of the players in the previous round, and this may motivate individuals to try and make their competitors look bad by preventing them from cooperating using antisocial punishment (i.e., spending a part of one’s monetary reward to reduce the monetary reward of another player). Pleasant and Barclay (2018) compared the groups using an unequal-variance *t* test and reported a statistically significant difference in the expected direction, $t(13.76) = 3.84$, $p =.002$, $d = 1.51$.

**Fig. 2:**
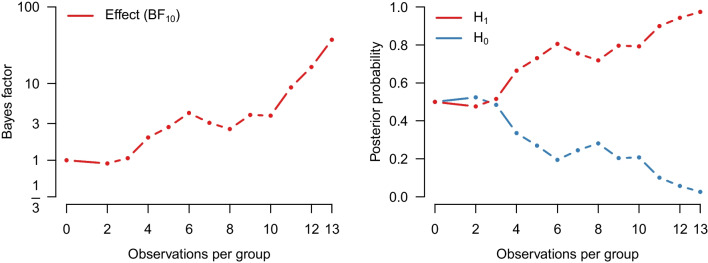
Results from a sequential Bayesian equal-variance *t* test applied to the data from Pleasant and Barclay (2018). The *left panel* shows the Bayes factor in favor of an effect and the *right panel* shows the probability of $\mathcal {H}_1$ and $\mathcal {H}_0$ as the data accumulate

**Fig. 3:**
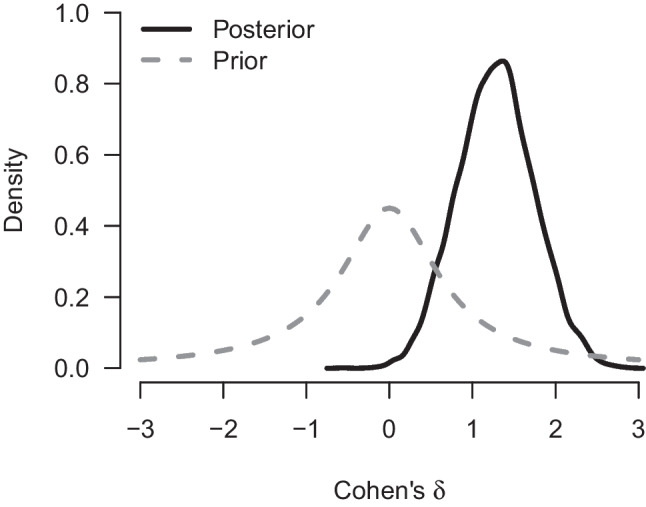
Prior and posterior distribution for Cohen’s $\delta$ for the Bayesian equal-variance *t* test under $\mathcal {H}_1$

We re-analyze the data using a Bayesian Student’s *t* test. Specifically, the data are assumed to come from two normal distributions with equal variance. The Bayes factor $\text {BF}_{10}$ quantifies the evidence that the data provide for the alternative hypothesis $\mathcal {H}_1: \delta \sim \text {Cauchy}(0, 1/\sqrt{2})$ over the null hypothesis $\mathcal {H}_1: \delta = 0$. For the purposes of illustration, we assume that Pleasant and Barclay (2018) analyzed their data sequentially – updating their beliefs every time after observing a new pair of groups from each condition. The left panel of Fig. 2 shows how the evidence for $\mathcal {H}_1$ accumulates over time whereas the right panel shows the associated flow of posterior probability for $\mathcal {H}_1$ and $\mathcal {H}_0$. After updating with all the data we find strong evidence in favor of the alternative hypothesis ($\text {BF}_{10 \text {Student}}$ = 37.2) with an associated posterior model probability for $\mathcal {H}_1$ of $37.2/38.2 \approx 0.974$ (assuming unit prior odds, i.e., a prior probability of $\nicefrac {1}{2}$). Figure 3 visualizes the prior and posterior distributions of the effect size $\delta$ under $\mathcal {H}_1$. The posterior mean equals $\delta = 1.28$, with a 95% credible interval ranging from 0.41 to 2.20.

## Bayesian Welch *t* test

While the equal-variance or Student’s *t* test is often the default approach in empirical papers, the variances are often unequal in practice (Delacre et al., 2017). Here we first extend the equal-variance case to the unequal-variance setting as outlined by Wetzels et al. (2009). The model specifications can be found in Appendix B.

### Prior distributions

In addition to the prior on the effect size, the Bayesian Welch *t* test also requires a suitable prior distribution on the “precision proportion” parameter ${\rho }$ (Dablander et al., 2020). This parameter $\rho$ denotes the proportion of the precision of the first group relative to the total precision, where precision is defined as the inverse of the variance. For instance, if the variances of the groups are 2 and 4, respectively, then the associated precisions are $\nicefrac {1}{2}$ and $\nicefrac {1}{4}$ yielding a total precision of $\nicefrac {3}{4}$. The corresponding $\rho$ is then the ratio of $\nicefrac {1}{2}$ to $\nicefrac {3}{4}$, thus, $\nicefrac {2}{3}$.

One reason for using the precision parametrization is that ${\rho }$ ranges from 0 to 1, allowing the convenient specification of a beta prior distribution. Here we assign ${\rho }$ an informed Beta(1.5, 1.5) prior distribution in which most mass is concentrated around values with realistic proportions.^7^ Since precision proportion does not provide an immediate intuition about the differences between the groups, we present the results in terms of the standard deviation ratio, $\text {SDR} = \sigma _1/\sigma _2$, which relates to the precision proportion ${\rho }$ as $\text {SDR} = \sqrt{\nicefrac {\rho }{(1-{\rho })}}$. The informed Beta(1.5, 1.5) prior on ${\rho }$ induces a prior on SDR that assigns approximately 90% probability mass to standard deviation ratios between $\nicefrac {1}{3}$ and 3.

### Running example (Continued)

**Fig. 4:**
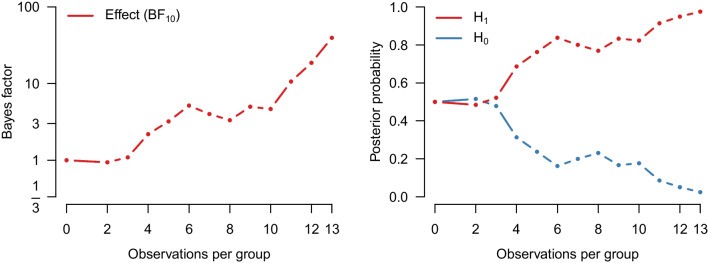
Results from a sequential Bayesian Welch *t* test applied to the data from Pleasant and Barclay (2018). The *left panel* shows the Bayes factor in favor of an effect and the *right panel* shows the probability of $\mathcal {H}_1$ and $\mathcal {H}_0$ as the data accumulate

**Fig. 5:**
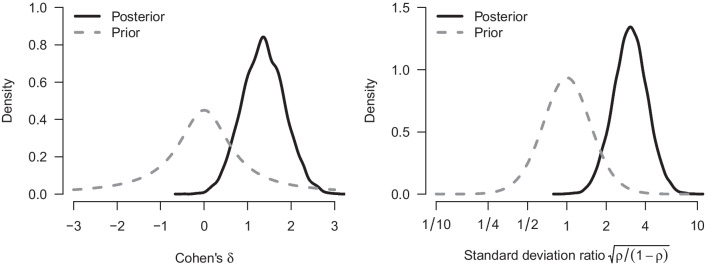
Prior and posterior distribution for Cohen’s $\delta$ and ${\rho }$ for the Bayesian Welch *t* test under $\mathcal {H}_1$. The *left panel* shows the prior and posterior distribution for $\delta$ under $\mathcal {H}_1$; the *right panel* shows the prior and posterior distribution for the standard deviation ratio (note the logarithmic scaling of *x*-axis)

We re-analyze the example from the previous section with our implementation of a Bayesian Welch *t* test. Figure 4 shows how our implementation of the Welch Bayes factor and posterior model probabilities progress sequentially. Using the prior distributions outlined above, the result after updating with all of the data indicates even stronger evidence in favor of an effect (i.e., in favor of a difference between means, $\text {BF}_{10, \text {Welch}} = 67.4$, compared to $\text {BF}_{10, \text {Student}} =37.2$ from the equal-variance test). The data are about 67.4 times more likely under the alternative hypothesis than under the null hypothesis, and this raises the probability for $\mathcal {H}_{1}$ from 0.5 to $67.4/68.4 = 0.99$. The left panel of Fig. 5 shows the associated prior and posterior distributions of the effect size $\delta$ under $\mathcal {H}_1$. The posterior mean equals $\delta = 1.36$ with a 95% CI that extends from 0.44 to 2.31. The right panel of Fig. 5 shows the prior and posterior distributions of the standard deviation ratio. The posterior mean of the standard deviation ratio equals $\text {SDR} = 3.20$ with a 95% credible interval ranging from 1.68 to 5.49; this confirms the visual impression from Fig. 1 that the standard deviation of the intervention group is substantially larger than that of the control group.

## Model-averaged Bayesian (MB) *t* test

In practice, it is usually not known with certainty whether or not the variances in the two groups are equal; in other words, researchers need to make inferences about the equality of means and the equality of variances at the same time.

A principled solution to this problem is provided by Bayesian model-averaging (Hinne et al., 2020; Hoeting et al., 1999), a methodology that avoids the standard two-step procedure (i.e., first test for equality of variance, then test for equality of means) by applying all pertinent models simultaneously, weighting their inferential impact with their plausibility.

Box 2 shows how to extend Bayes factors to *inclusion* Bayes factors when comparing sets of models. The inclusion factor generalizes the Bayes factor by dividing the posterior odds by the prior odds for sets of models rather than individual models.

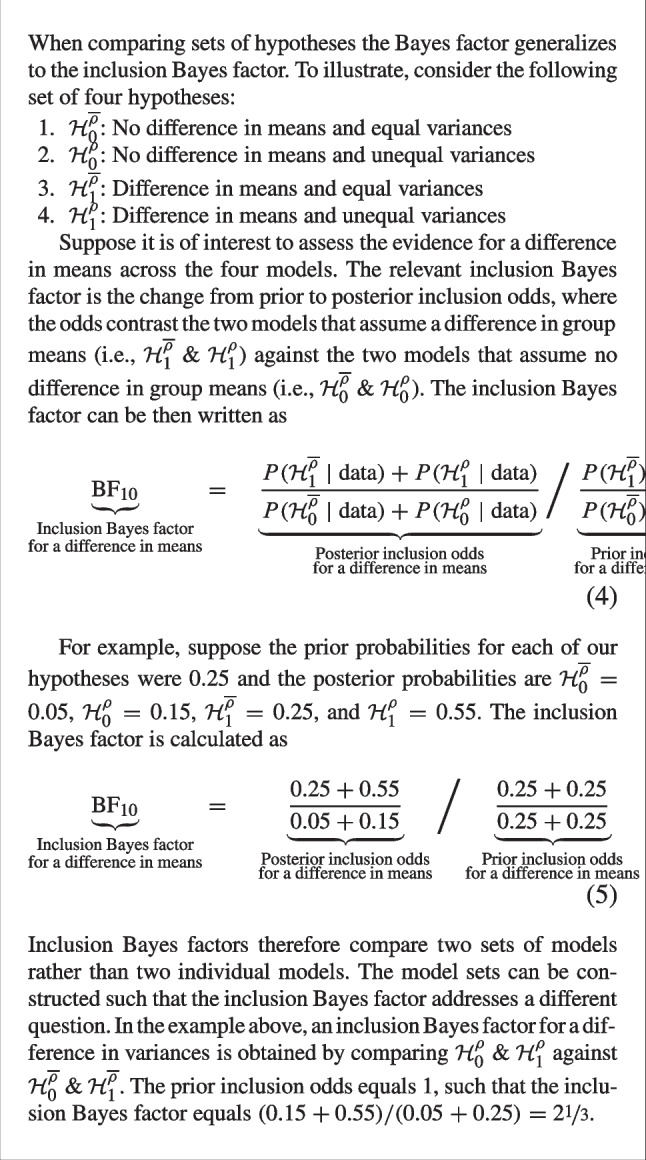



When the data strongly favor equal-variance models, the inclusion Bayes factor from InfoBox 2 approximates the equal-variance *t* test; when the data strongly favor unequal-variance models, the inclusion Bayes factor approximates the unequal-variance *t* test. When the data do not provide strong support concerning equality of variances, the inclusion Bayes factor is affected both by equal-variance models and by unequal-variance models.

Bayesian model-averaged parameter estimation allows us to account for the uncertainty of each model. Specifically, the model-averaged posterior distribution is a weighted average of the posterior distributions from each of the models in the ensemble, with the mixing weight given by each model’s posterior probability. Algorithmically, one may construct the model-averaged posterior distribution as follows: (1) sample a model in proportion to the posterior model probabilities; (2) from the selected model, draw a parameter value from its posterior distribution; (3) repeat steps 1 and 2 many times.

**Fig. 6:**
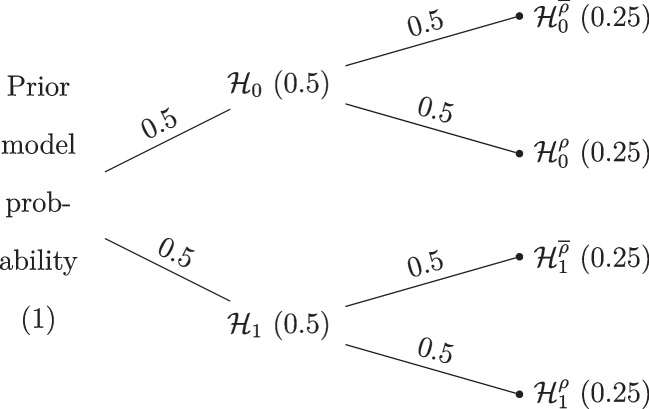
Default prior model probabilities of the model-averaged Bayesian *t* test. Marginal model probabilities are displayed on the nodes and conditional model probabilities are displayed on the edges

Figure 6 shows how the model space is partitioned in our model-averaged Bayesian *t* test (MB *t* test). We see that the prior model probability is divided equally across models assuming equal and unequal means as well as across models assuming equal and unequal variances. The prior distributions for the unequal-variance *t* test are the same as in the previous section. The equal-variance models are defined by ${\rho } = 0.5$. The prior distribution on the standardized mean difference is again Cauchy(0, 1/$\sqrt{2}$).

### Running example (Continued)

**Fig. 7:**
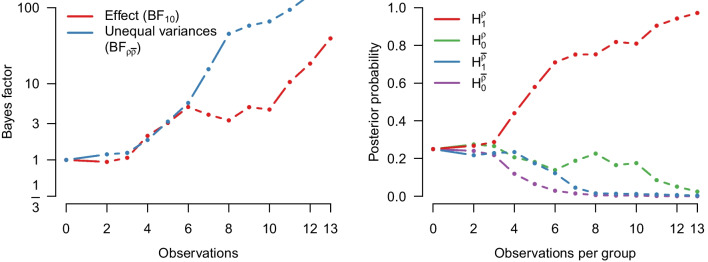
Results from a sequential model-averaged Bayesian *t* test applied to the data from Pleasant and Barclay (2018). The *left panel* shows the inclusion Bayes factor in favor of a difference in means and in favor of unequal variances. The *right panel* shows the probability of the four different models as the data accumulate. Note that the last two Bayes factors in favor of unequal variances are 147.33 and 256.00 and therefore outside the plotting range

We now analyze the running example using the MB *t* test by model-averaging over equal and unequal variances. The left panel of Fig. 7 shows the inclusion Bayes factor for equal and unequal variances, whereas the right panel of Fig. 7 tracks the probability of the four different models over time. Specifically, the left panel shows that the evidence for an effect (red line) increases as the data accumulate, up to a final value of $\text {BF}_{10, \text {MB}}$ = 39.5. This panel also shows that the evidence for unequal variances (blue line) increases as the data accumulate, with a final value of ($\text {BF}_{{\rho }\overline{\rho }, \text {MB}}$ = 265.6).

The right panel of Fig. 7 shows that the final posterior probability is highest for the model that assumes a difference in both means and variances (red line; $\mathcal {H}_1^{\rho }$ = 0.98); the next best model assumes no difference in means but a difference in variances (green line) – it remains a non-negligible competitor until almost all data have been observed.

**Fig. 8:**
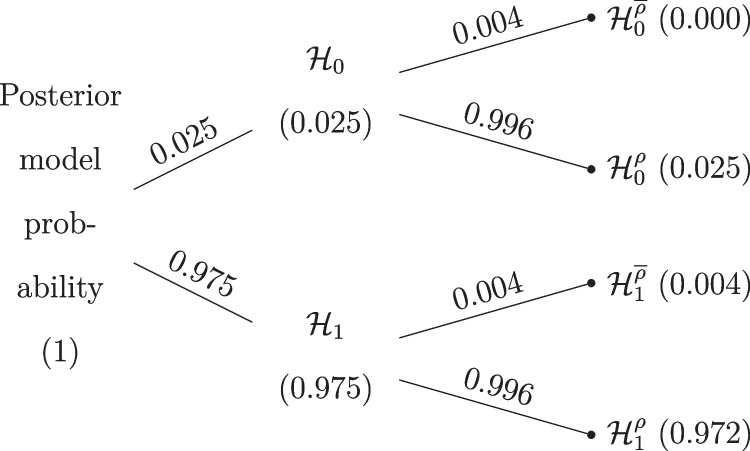
Posterior model probabilities of the model-averaged Bayesian *t* test applied to the data from Pleasant and Barclay (2018). Total probabilities are displayed on the nodes and conditional probabilities on the edges. $\mathcal {H}_0$ denotes the models assuming the null hypotheses to be true, $\mathcal {H}_1$ denotes the models assuming the alternative hypotheses to be true; $\mathcal {H}^{\overline{\rho }}$ denotes equal-variance models, and $\mathcal {H}^{\rho }$ denotes the unequal-variance models

Figure 8 shows the posterior model probabilities after all the data are analyzed. We see that most of the posterior model probability is concentrated on the $\mathcal {H}_1^{\rho }$ model.

**Fig. 9:**
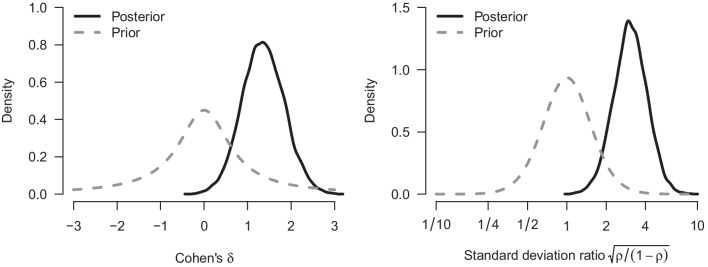
Prior and posterior distribution for Cohen’s $\delta$ and ${\rho }$ for the Bayesian model-averaged *t* test. The *left panel* shows the conditional prior and posterior distribution for $\delta$ assuming an effect to be present; the *right panel* shows the conditional prior and posterior distribution for the standard deviation ratio assuming unequal variance (note the logarithmic scaling of *x*-axis)

With respect to parameter estimation, the left panel of Fig. 9 shows the model-averaged prior and posterior distribution for the difference in means $\delta$. The model-averaged posterior mean equals $\delta = 1.32$ and the central 95% CI ranges from 0.00 to 2.31. The right panel of Fig. 9 shows the model-averaged prior and posterior distributions of Cohen’s $\delta$ and SDR across the models in which the parameters are present. The model-averaged posterior mean equals SDR = 2.84 and the central 95% CI ranges from 1.67 to 5.41, suggesting a pronounced difference in standard deviations between the two groups, consistent with the visual impression from Fig. 1. The standard deviation in the intervention condition is about three times larger than that in the control condition.

## Robust model-averaged Bayesian (RoMB) *t* test

**Fig. 10:**
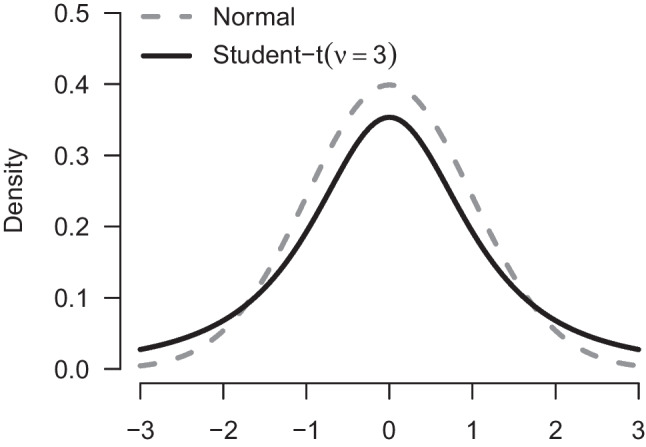
The *t*-distribution has thicker tails than the normal distribution

A limitation of model-averaging over the four models in the previous section is that all of these models use a normal likelihood. These models are therefore sensitive to rare extreme observations. We mitigate this weakness by also model-averaging over *t*-likelihoods, resulting in a Robust Model-Averaged Bayesian (RoMB) *t* test. Figure 10 illustrates why extreme observations are more common under *t*-distributions. The normal distribution (grey) has much thinner tails than a *t*-distribution with three degrees of freedom (black). Therefore, when using a normal likelihood, the estimate will be strongly influenced by outliers compared to the *t*-distribution, under which extreme observations are much less surprising. In addition to accommodating outliers, Bayesian model-averaging also allows a test for the presence versus absence of outliers, namely an inclusion Bayes factor that contrasts the set of models with a *t*-likelihood to the set of models with a normal likelihood.

**Fig. 11:**
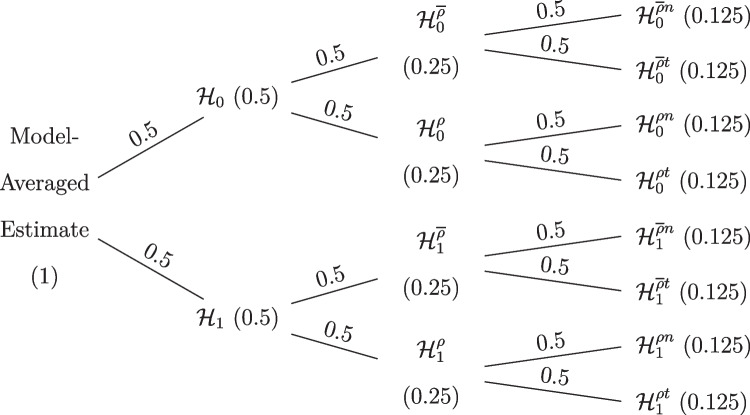
Prior model probabilities of the robust model-averaged *t* test. Marginal model probabilities are displayed on the nodes and conditional model probabilities on the edges. $\mathcal {H}_0$ denotes the models assuming the null hypotheses of equal means to be true, $\mathcal {H}_1$ denotes the models assuming the alternative hypotheses to be true. $\mathcal {H}^{\overline{\rho }}$ denotes equal-variance models, $\mathcal {H}^{\rho }$ denotes the unequal-variance models. $\mathcal {H}^{t}$ denotes the models using *t*-likelihoods and $\mathcal {H}^{n}$ denotes the models using normal likelihood

Figure 11 shows the prior model space. Compared to the models from the previous section, the model space has now been extended by including *t*-likelihood models. Model-averaged inference is now based on $2\times 2\times 2 = 8$ models simultaneously and the prior model probability is distributed equally across the different models.

### Prior distributions

We again specify a Cauchy(0, 1/$\sqrt{2}$) prior distribution for the effect size $\delta$ and a Beta(1.5, 1.5) prior distribution for the precision proportion ${\rho }$. Inclusion of the *t*-likelihood requires specification of one additional prior distribution – a prior distribution for the parameter that indicates the degrees of freedom of the *t*-distribution. We use an exponential distribution with scale 1 shifted to the right by two ($\nu \sim e^{-(x-2)}$), resulting in a prior mean of three degrees of freedom and interquartile range from 2.3 to 3.4 degrees of freedom. By shifting the prior distribution, we ensure that the mean and variance of the *t*-likelihood are always defined, which is essential for the effect size parametrization in terms of Cohen’s $\delta$. The shifted exponential prior distribution assigns most of the mass to low degrees of freedom, which makes it sufficiently distinct from the models using the normal likelihood to allow for a diagnostic test of outliers.

### Running example (Continued)

**Fig. 12:**
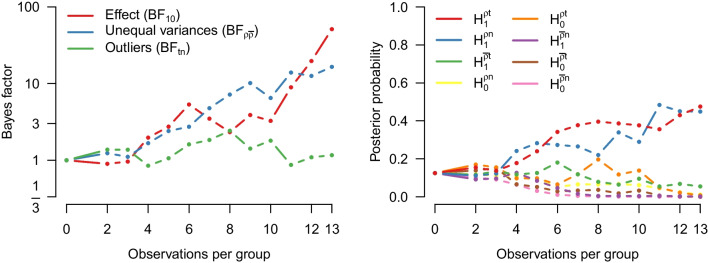
Results from a sequential robust model-averaged Bayesian *t* test applied to the data from Pleasant and Barclay (2018). The *left panel* shows the inclusion Bayes factor in favor of an effect, unequal variances, and outliers. The *right panel* shows the probability of the eight different models as the data accumulate

**Fig. 13:**
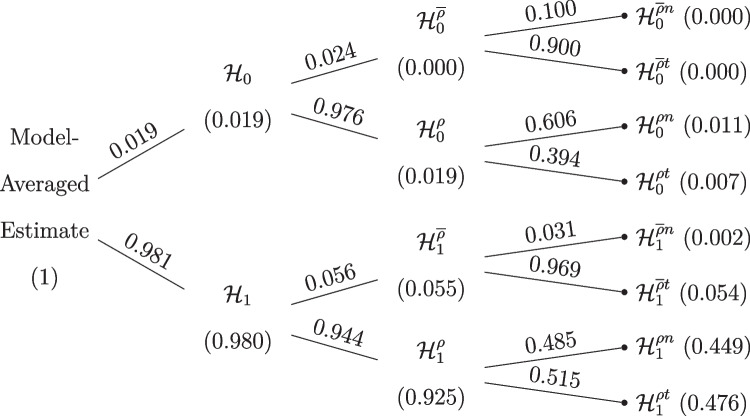
Posterior model probabilities of the robust model-averaged Bayesian *t* test applied to the data from Pleasant and Barclay (2018). Total probabilities are displayed on the nodes and conditional probabilities on the edges. $\mathcal {H}_0$ denotes the models assuming the null hypotheses to be true, $\mathcal {H}_1$ denotes the models assuming the alternative hypotheses to be true. $\mathcal {H}^{\overline{\rho }}$ denotes equal-variance models, $\mathcal {H}^{\rho }$ denotes the unequal-variance models. $\mathcal {H}^{t}$ denotes the models using *t*-likelihoods and $\mathcal {H}^{n}$ denotes the models using normal likelihood

We now illustrate model-averaging over *t*-likelihoods with the example from the previous sections. The left panel of Fig. 12 shows the sequential inclusion Bayes factors for a difference in means (red line), unequal variances (blue line), and outliers (green line). The evidence for the presence of a difference in means has become somewhat stronger now that *t*-likelihoods are also considered ($\text {BF}_{10, \text {RoMB}}$ = 51.2, up from 39.5 based on only normal-likelihood models). In addition, there is absence of evidence regarding outliers ($\text {BF}^{tn}$ = 1.16) and strong evidence for unequal variances ($\text {BF}^{{\rho }\overline{\rho }}$ = 16.9, down from 265.6 based on only normal-likelihood models). The corresponding posterior probabilities can be found in Fig. 13.

**Fig. 14:**
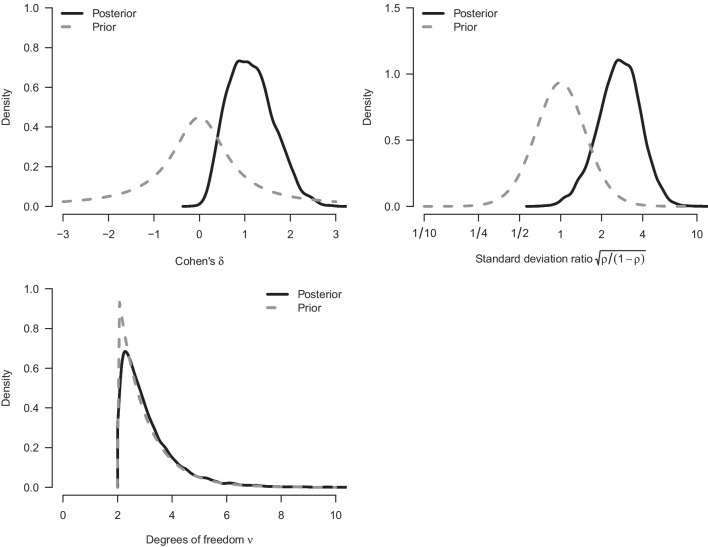
Prior and posterior distribution for Cohen’s $\delta$, ${\rho }$, and *t* for the robust model-averaged Bayesian *t* test. All panels show the conditional prior and posterior distributions assuming the parameter to be present

Figure 14 shows the posterior distribution of the parameters model-averaged across the models in which the parameters are present. The posterior mean for effect size is $\delta = 1.12$, 95% CI [0.18, 2.19]. Note that the posterior mean is somewhat smaller but more precise than the one obtained without accounting for outliers. The model-averaged posterior mean for SDR equals 2.75, 95% CI [1.00, 5.20]. For the degrees of freedom, the model-averaged posterior median equals $\nu = 2.80$, 95% CI [2.05 5.92]. Note that in this section we tested a sharp point null hypothesis; however, researchers who believe that the point null is never true can conduct perinull testing instead, as outlined in Appendix A (cf. Ly and Wagenmakers, in press).

## Sequential updating with the robust model-averaged Bayesian *t* test

In order to incorporate domain expertise or knowledge obtained from previous studies, researchers may wish to use prior distributions that are more informed than the default priors applied above (Gronau et al., 2020; Stefan et al., 2020, 2019). One straightforward example is that researchers who conduct a replication study may wish to use the posterior distribution from the original study as the prior distribution for the analysis of the replication data. In this procedure, known as the replication Bayes factor, usually only the prior distribution will be updated to correspond to the posterior distribution of the previous study (Verhagen & Wagenmakers, 2014). In the model-averaging case, we need to extend this by also updating the prior model-probabilities to correspond to the posterior model probabilities of the original study. Note that this is a key benefit of model-averaging – in the model selection case, the sequential inference becomes compromised if researchers need to switch between different types of tests during the updating process because the evidence for different models has changed. Ly et al. (2019) show that the replication Bayes factor can be obtained by dividing the Bayes factor based on analyzing both data sets together (original study and replication) by the Bayes factor from the original study, which is the approach that we employ here (as it facilitates updating on the nuisance priors on the variances within each group, which cannot be specified manually in our software implementation).

### Running example

Assume a group of scientists conducted a replication of the Pleasant and Barclay (2018) study using the RoMB *t* test. They collected 20 people in the treatment group (mean = 12.35, SD = 12.18) and 20 people in the control group (mean = 3.65, SD = 3.94).

Applying RoMB to the complete data set (the original example and this replication), we find $\text {BF}_{10, \text {RoMB}}$ = 1135.2, $\text {BF}_{{\rho }\overline{\rho }, \text {RoMB}}$ = 30370.9, and $\text {BF}_{{\nu }\overline{\nu }, \text {RoMB}}$ = 0.187. Thus, the replication Bayes factors from evidence updating ($\nicefrac {\text {BF}_{combined}}{\text {BF}_{original}}$) are $\text {BF}_{10, \text {rep}}$ = 22.2 (1135.2/51.2), $\text {BF}_{{\rho }\overline{\rho }, \text {rep}}$ = 1797.1 (30370.9/16.9), and $\text {BF}_{{\nu }\overline{\nu }, \text {rep}}$ = 0.161 (0.187/1.16). In other words, we would conclude that the data of the replication study are more in line with the difference in means being the same as in the first study then with the null hypothesis of no difference. For the equality of variances, we also conclude that the data is more in line with the results of the first study than with the null of equal variances. However, for the outliers, we see stronger evidence for the absence of outliers than for the alternative defined by the posterior of the first study.

The total sample size (original study and replication study) model-averaged estimates are $\delta = 1.07$, 95% CI [0.48, 1.64], $\rho = 0.90$, 95% CI [0.81, 0.95], and median $\nu = \infty$, 95% CI $[2.57, \infty ]$. These estimates underscore the results from the Bayes factor analysis: the data indicates a large difference in means and unequal variances but absence of outliers. In conclusion, the hypothetical replication study would support the original findings about the presence of the effect and unequal variances, and additionally provide evidence for the absence of outliers.

## Additional example: impact of accuracy nudge interventions on discernment

While the model-averaged Bayesian *t* tests allow for more rich and robust inferences on the running example of Pleasant and Barclay (2018), the conclusions regarding the mean difference remained similar regardless of the type of test used. In this section, we introduce an additional example, which underscores how the new model-averaged *t* tests can lead to substantially different conclusions for widely used behavioral interventions. Roozenbeek et al. (2021) conducted a study to test the effectiveness of an accuracy nudge intervention. This study was a direct preregistered replication of Pennycook et al. (2020).^8^ The intervention involved asking participants the question, “To the best of your knowledge, is the above headline accurate?”.

**Fig. 15:**
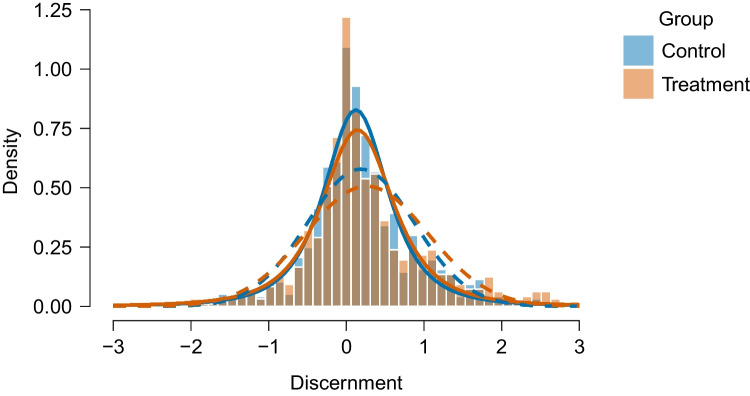
Discernment scores for the accuracy nudge treatment and control condition are better captured by a *t*-distribution than a normal distribution. *t*-distributions are displayed as *full lines* and normal distributions as *dashed lines*. Data from Roozenbeek et al. (2021)

**Table 1:** Frequentist and Bayesian *t* tests can lead to different conclusions

*t* test	Prior	Result	Conclusion
(Frequentist) Welch’s	Not applicable	$p = 0.044$	Reject $\mathcal {H}_0$
Bayesian model-averaged	Default	$\text {BF}_{10, \text {MB}}$ = 0.419	Anecdotal evidence for $\mathcal {H}_0$
Robust Bayesian model-averaged	Default	$\text {BF}_{10, \text {RoMB}}$ = 0.036	Strong evidence for $\mathcal {H}_0$
Bayesian model-averaged	Informed	$\text {BF}_{10, \text {MB}}$ = 1.70	Anecdotal evidence for $\mathcal {H}_1$
Robust Bayesian model-averaged	Informed	$\text {BF}_{10, \text {RoMB}}$ = 0.399	Anecdotal evidence for $\mathcal {H}_0$

*Note.* Data from Roozenbeek et al. (2021). The default prior corresponds to $\delta \sim \text {Cauchy}(0, 1/\sqrt{(}2))$, the informed prior corresponds to $\delta \sim \text {Cauchy}(0, 0.05)$

Roozenbeek et al. (2021) tested whether this nudge increased discernment, which is defined as the difference in sharing intentions for real versus false headlines. In other words, people with high discernment can differentiate between true and false headlines well, while those with low discernment struggle to do so. Each participant rated 15 real and 15 false headlines related to COVID-19 in a random order and was asked the following question: “If you were to see the above on social media, how likely would you be to share it?” (from extremely unlikely to extremely likely on a six-point Likert scale). Roozenbeek et al. (2021) then compared the accuracy nudge group to a simple control group using a participant-level *t* test of the difference score between sharing intentions for true vs. false headlines (i.e., discernment). This “yielded a significant (noncorrected) effect for discernment (treatment: $\text {M} = 0.26$, control: $\text {M} = 0.19$, mean difference: $-0.075, 95\% \text {CI} = [-0.15, -0.0019]), t(1581) = -2.013, p =.044, d = -0.10, 95\% \text {CI} = [-0.20, -0.0025]$. A Bayesian *t* test revealed a Bayes factor (BF) indicating that the data are approximately 1.7 times more likely to occur under the focal hypothesis than under the null hypothesis ($\text {BF}_{10} = 1.705$).” (Roozenbeek et al., 2021, p.173). Roozenbeek et al. (2021) use a very tight Cauchy(0, 0.05) prior incorporating their expectation of small effects based on the target study. We first reanalyze the paper with the default priors of our *t* test and then consider how the results are affected by switching to the informed prior used by Roozenbeek et al. (2021).

The model-averaged Bayesian *t* test (i.e., only averaging over the equal and unequal variance versions of the test) finds weak or anecdotal evidence against an effect, $\text {BF}_{10, \text {MB}}$ = 0.419, and strong evidence for unequal variances $\text {BF}_{{\rho }\overline{\rho }, \text {MB}}$ = 62.1, a result similar to Roozenbeek et al. (2021). The model-averaged estimates are $\delta = 0.03$, 95% CI [0.00, 0.17], $\rho = 0.57$, 95% CI [0.52, 0.60] corresponding to a minuscule effect and modest inequality in variances.

Figure 15 visualizes the discernment scores in each group with overlaying normal (dashed lines) and *t*-distribution (full lines) from the posterior means of models assuming the presence of the effect and unequal variances. The visualization of the fit indicates that the *t*-distributions can accommodate the data much better than the normal distributions, which does not capture the data in the tails well. We therefore next revisit the data using the robust version of our *t* test.^9^

The robust version of the *t* test finds strong evidence for the absence of a mean difference ($\text {BF}_{10, \text {RoMB}}$ = 0.036), weak evidence against unequal variances ($\text {BF}_{{\rho }\overline{\rho }, \text {RoMB}}$ = 0.529), and strong evidence for outliers ($\text {BF}_{{\nu }\overline{\nu }, \text {RoMB}} = 1.4 \times 10^{38}$). The model-averaged effect size estimate shrinks to zero, $\delta = 0.00$, 95% CI [0.00, 0.00], and the inequality in variances almost disappears $\rho = 0.52$, 95% CI [0.50, 0.59]. The degrees of freedom approach the lower limit, median $\nu = 2.52$, 95% CI [2.12, 3.09], indicating that the distributions have very heavy tails. In other words, when taking outliers into account, the evidence for a mean difference between conditions changes from weak evidence against an effect to strong evidence against an effect.

Finally, when we use the informed Cauchy(0, 0.05) prior by Roozenbeek et al. (2021) we find weak evidence in favor of the null hypothesis, $\text {BF}_{10, \text {RoMB}}$ = 0.399, (in contrast to Roozenbeek et al., 2021 who found weak evidence for the alternative hypothesis even under same prior distributions), weak evidence against unequal variances $\text {BF}_{{\rho }\overline{\rho }, \text {RoMB}}$ = 0.547, and still extreme evidence for outliers $\text {BF}_{{\nu }\overline{\nu }, \text {RoMB}} = 1.0 \times 10^{38}$). The model-averaged estimates are $\delta = 0.00$, 95% CI $[-0.02, 0.05]$, $\rho = 0.52$, 95% CI [0.50, 0.59], and median $\nu = 2.53$, 95% CI [2.14, 3.10].

The example shows how model-averaging over *t*-likeli-hoods can substantially change the conclusions in typical psychology experiments when the effects are driven by extreme values (Table 1). This underscores the importance of relying on the RoMB version of the *t* test for most applications as it will substantially increase robustness when outliers are present but come at little cost when they are absent (as in this case, the most weight will be given to the models assuming the absence of outliers). Note that we do not take this example to refute the effectiveness of accuracy nudges in general, as this would require a broader reanalysis of all relevant papers (e.g., Martel et al., 2024; Pennycook et al., 2020) using meta-analytic techniques, which is outside the scope of the current manuscript.

## Simulation

To assess parameter recovery and the benefits of the model-averaging methods, we evaluated the performance of the proposed methods via a simulation study. We orthogonally varied the following four factors:

- Effect size (Cohen’s $\delta$): 0, 0.3, 0.5;
- Standard deviation ratio: 1, 1.5, 2;
- Presence of outliers by simulating from a normal distribution (i.e., no outliers) or from Student’s *t*-distribution with $\nu = \{10,5\}$ degrees of freedom;
- Total sample size: 20, 50, 100.
- Sample size allocation: $\nicefrac {1}{2}$, 1, 2

Each setting in this $3 \times 3 \times 3 \times 3 \times 3$ design was used to simulate 1,000 fictitious experiments. We compared the performance of the following four tests, outlined above: (1) the Bayesian version of Student’s *t* test; (2) the Bayesian version of the Welch *t* test; (3) the model-averaged version of *t* test that incorporates uncertainty about equality of variances (MB *t* test); and (4) the model-averaged version that further incorporates uncertainty about outliers (RoMB *t* test). We evaluate the impact of model specification on the Bayes factors in terms of the evidence distortion factor. The evidence distortion factor allows us to evaluate the change in evidence from applying an incorrect model (i.e., a model with a likelihood that does not correspond to the data-generating process). For example, suppose data were simulated from a model corresponding to Student’s *t* test. If we were to use Welch *t* test, the relative evidence distortion factor (i.e., $\text {EDF(Welch/Student)}$) would correspond to $\text {EDF} = \nicefrac {\text {BF}_\text {10, Welch}}{\text {BF}_\text {10, Student}}$. Similarly, the evidence distortion factor EDF(MB/Student) = $\nicefrac {\text {BF}_{10, \text {MB}}}{ \text {BF}_{10, \text {Student}}}$ measure the distortion of evidence if a normal likelihood equal/unequal model-averaged *t* test is used instead. Consequently, using the correct test for a given data set corresponds to an evidence distortion factor of 1, i.e., no evidence distortion. For brevity, we discuss results from a few selected conditions; a detailed summary of the complete factorial design can be reproduced at https://osf.io/mwkp6/.

**Fig. 16:**
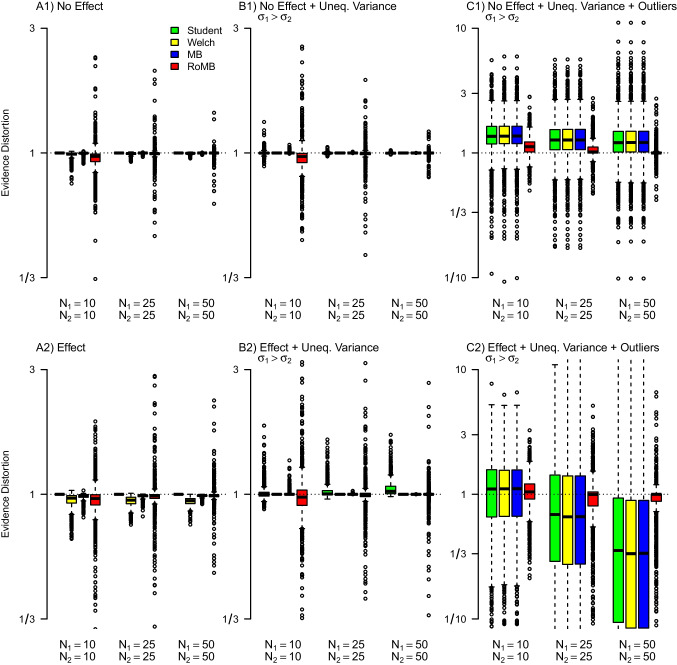
Evidence distortion of the Bayes factor for the difference in means for different methods and conditions under equal sample sizes. The four methods are the Student’s *t* test (*green*), the Welch *t* test (*yellow*), a model-averaged version of *t* test that combines Student’s and Welch’s *t* test (MB *t* test; *blue*) and a version that also incorporates uncertainty about the outliers (RoMB *t* test; *red*). Whenever the difference in means is present (second row) then $\delta$ = 0.5. Whenever the variances are unequal (columns 2 and 3) SDR is 2, whenever the data are simulated from a *t*-distribution (column 3) this was done with $\nu$ = 5 degrees of freedom

**Fig. 17:**
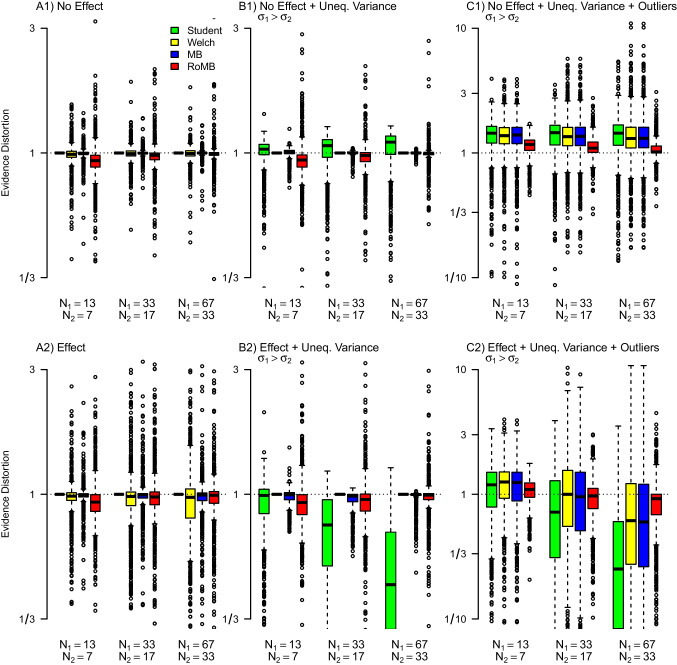
Evidence distortion of the Bayes factor for the difference in means for different methods and conditions under unequal sample sizes. The four methods are the Student’s *t* test (*green*), the Welch’s *t* test (*yellow*), a model-averaged version of *t* test that combines Student’s and Welch’s *t* test (MB *t* test; *blue*) and a version that also incorporates uncertainty about the outliers (RoMB *t* test; *red*). Whenever the difference in means is present (second row) then $\delta$ = 0.5. Whenever the variances are unequal (columns 2 and 3) SDR is 2, whenever the data are simulated from a *t*-distribution (column 3) this was done with $\nu$ = 5 degrees of freedom.

### Equal sample sizes

Figure 16 shows a pronounced effect on the evidence distortion factor for Student’s, Welch’s, and MB *t* test in the presence of outliers for equal sample sizes in each group (panel C1 and C2). Specifically, both Student’s and Welch’s versions of the Bayesian *t* test tend to overestimate the evidence in favor of the alternative hypothesis when the variances are unequal, outliers are present, and there is no difference in the means (C1); in addition, these versions underestimate the evidence for the alternative hypothesis when the variances are unequal, outliers are present, and there is a difference in the means (C2). In other words, when outliers are present but unaccounted for, this makes it more difficult to identify the correct model for the group means. Moreover, the underestimation of evidence was rapidly increasing with sample size as can be seen by the increasing distortion with increasing sample size in C2. However, even the model-averaged test occasionally leads to overestimation or underestimation in comparison to the true model. The reason is that sometimes when simulating from the *t*-likelihoods, we do not actually observe any extreme observations (i.e., the actual data is better described by normal), in which case the RoMB does not select the *t*-likelihood models. In the cases reported on the OSF, we see that while the model-averaged version of the *t* test produced slightly more variable estimates, it was always centered around the correct value and provided appropriate evidence assessment even in conditions with unequal variances or outliers.

In line with previous research (Lumley et al., 2002) the difference between the methods in terms of the posterior mean effect size was relatively modest. (Appendix C). Overall, averaging over *t*-likelihoods comes with small costs when outliers are absent, but with large gains in particular for testing when outliers are present.

### Unequal sample sizes

Figure 17 shows how unequal sample sizes can further exacerbate the evidence distortion factor when Student’s *t* test is used. Specifically, we can see that when sample sizes and variances are unequal, the Bayesian Student’s *t* test shows much stronger evidence distortion than under equal sample sizes (panel B2). However, as in Fig. 16, applying the RoMB results in only minimally increased variability when sample sizes are equal, or no outliers are present (columns A and B) but has a substantial benefit when outliers are present (column C). Appendix D shows the same Figure when the larger variance is in the smaller group. This shows a similar pattern, however, in the presence of an effect, the inflation is inverted with the Student’s *t* test overestimating rather than underestimating the evidence. The next section analyses the difference in evidence between Welch’s and Student’s *t* test for a wider range of effect sizes and variance ratios.

**Fig. 18:**
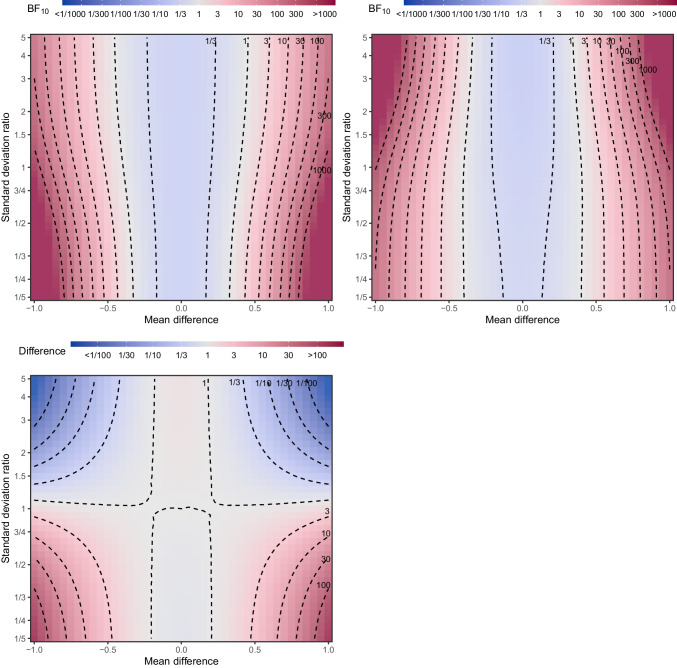
Comparison of Bayes factors from Bayesian Student’s *t* test and Bayesian Welch *t* test on a sample with unequal sample size. *Top left*: Bayesian Student’s *t* test; *top right*: Bayesian Welch *t* test; *bottom left*: ratio of evidence from the two tests (log scaled). Positive mean difference and standard deviation ratios larger than one correspond to larger means and standard deviations in the larger sample group

## Comparing Student’s and Welch’s *t* tests under unequal sample sizes for a range of effect sizes and standard deviation ratios

This section highlights further how Student and Welch *t* tests can indicate considerably different evidence for the alternative under unequal sample sizes. In particular, we show how the evidence differs between Bayesian Student’s *t* test and Bayesian Welch’s *t* test for different observed mean differences and standard deviation ratios.

Both Bayesian Student’s *t* test and Bayesian Welch’s *t* test can be computed from summary statistics: the sample means, standard deviations, and sample size. This allows researchers to conveniently compute the evidence for a range of possible observed mean differences and variance ratios under each test and compare the results. While both tests perform almost identically when sample sizes are equal, the evidence in favor of the alternative vs the null hypothesis can differ drastically when the sample sizes differ.

Figure 18 visualizes the evidence (log scaled) in favor of the alternative hypothesis from the Bayesian Student *t* test (first row left), Bayesian Welch *t* test (first row right), and the ratio of evidence from the two tests (log scaled, second row left), under a range of mean differences (MD, *x*-axis), standard deviation ratios (SDR, *y* axis), and with unequal sample sizes ($n_1 = 33$, $n_2 = 66$). We fix the grand mean ($\mu$) to zero and the grand standard deviation ($\sigma$) to one. In the figure, positive mean difference and standard deviation ratios larger than one correspond to larger means and standard deviations in the larger sample group, i.e., $\mu _1 = \mu - 0.5\text {MD}, \mu _2 = \mu + 0.5\text {MD}, \sigma _1 = \sqrt{(2 \sigma ^2 \text {SDR}^{-2} / (1/\text {SDR} + 1))}, \sigma _2 = \sqrt{(2 * \sigma ^2 * \text {SDR}^2 / (\text {SDR}^2 + 1))}$.

The Bayesian Welch *t* test (the upper right panel), i.e., the correct test in cases when the true standard deviation ratio differs from one, shows the evidence in favor of the alternative hypothesis is larger at the same mean differences with increasing standard deviation in the larger group (the upper half of the figure) in comparison to increasing standard deviation in the smaller group. This is the appropriate behavior as larger sample size in the more variable group increases our certainty about differences between the two groups more than larger sample size in the less variable group.

The Bayesian Student *t* test (the upper left panel) shows exactly the opposite pattern – the evidence in favor of the alternative hypothesis is smaller at the same mean differences with increasing standard deviation in the larger group (the upper half of the figure) in comparison to increasing standard deviation in the smaller group. This inappropriate behavior results from the larger sample size in the less variable group artificially increasing certainty about the differences between the two groups if the difference in the variances between the groups is ignored.

The bottom right panel with the difference between the evidence from the Bayesian Student’s *t* test and Bayesian Welch’s *t* test highlights that the evidence can increase more than hundred-fold when considering mean differences of about 1 standard deviation ratios of 5, but even smaller mean differences and standard deviation ratio can easily lead to doubling of the evidence.

## Concluding comments

We introduced a model-averaged Bayesian *t* test that consists of the following ensemble of eight models: (1) models assuming a difference in group means is absent vs. present; (2) models assuming between-group variances are equal vs. unequal; and (3) models assuming likelihoods are normal vs. based on the *t*-distribution. A key advantage of this methodology is that it obviates the need to test assumptions sequentially and then base inference on a single model selected in an all-or-none fashion. With the model-averaged Bayesian *t* test, researchers can focus their attention on the substantive research questions rather than the statistical analysis plan, as the data will guide the inference to be based most strongly on the models that predict the data best. Our simulations show that the benefits of model-averaging are especially pronounced for Bayesian testing, whereas the improvements in performance for estimation are relatively modest.

Our simulation study and the example of Roozenbeek et al. (2021) show that for realistic settings, choosing the wrong type of *t* test can lead to a sizeable distortion in evidence. Given that using the RoMB *t* test usually comes at little cost when a simpler model describes that data best but has substantial benefits for testing when unequal variances or outliers are present, we believe it is promising tool in the toolbox of approaches for comparing independent group means.

The ensemble method is less dependent on particular assumptions (e.g., equality of variances, normal likelihood) than any particular model in the ensemble; nevertheless, the ensemble method is not assumption-free. Specifically, the method assumes that at least one of the specified models provides an adequate description of the data. Sometimes this might not be the case. Especially the *t*-likelihood might not be able to capture many of the ways in which outliers operate in practice. First, the *t*-likelihood assumes that outliers are symmetric. Second, the maximum spread of the *t*-distribution might be too small to accommodate more variable data-generating processes, such as discrete mixtures of distributions. While we believe that even in this case the *t*-likelihood will still provide an improvement over simply using a normal likelihood, it is important to also consider non-parametric or rank tests (e.g., MacFarland & Yates, 2016).

In addition, our test is not suitable for all data types. Our *t* test is based on a linear link function with normal likelihood. Different data types require different link or likelihood functions. For example, proportion data may be analyzed with Bayesian binomial test or contingency tables (Morey & Rouder, 2018; Wagenmakers & Grünwald, 2006; Jamil et al., 2017) and probability judgments using a beta model (Ferrari & Cribari-Neto, 2004). In addition, Likert scale data may be better analyzed with cumulative link models specifically designed for such data (McElreath, 2020; Bürkner and Vuorre, 2019, pp. 394–410) rather than with any of the model-averaged Bayesian *t* tests presented in this manuscript.

One pragmatic disadvantage of Bayesian model-averaging is the increased computation time. However, the time penalty is not prohibitive; moreover, for model-averaging across models with normal likelihood the computation time is independent of sample size (since the models can be estimated with summary statistics). With *t*-likelihoods the estimation time does increase with sample size; however, the time penalty remains modest.^10^

The Bayesian model-averaged *t* test can be expanded in several ways. For example, lognormal and gamma likelihood functions could be considered in case of positively bounded values. The set-up could be also generalized from two-sample to multigroup settings, allowing researchers to draw robust and reliable inference for ANOVA-like problems under differences in variances or in the presence of outliers.

To facilitate the application of the proposed methodology in empirical practice we implemented the robust Bayesian *t* test in the RoBTT R package with accompanying vignettes as well as the graphical user interface statistical software JASP (JASP Team, 2022). We also provide a tutorial video for the JASP implementation at https://www.youtube.com/watch?v=EcuzGTIcorQ

We do not subscribe to the sentiment that all interesting scientific questions in psychology can be broken down into two groups and a Robust Bayesian model-averaged *t* test; nevertheless, the comparison of two group means represents one of the oldest and most popular inferential scenarios, and we hope that, when compared to what is now standard practice, the methodology proposed here can help experimental psychologists draw conclusions that are richer and more robust.

### Open practices and data availability

All data and materials to reproduce the analyses in this article are available at https://osf.io/mwkp6/.

## Figures and Tables

**Fig. 19:**
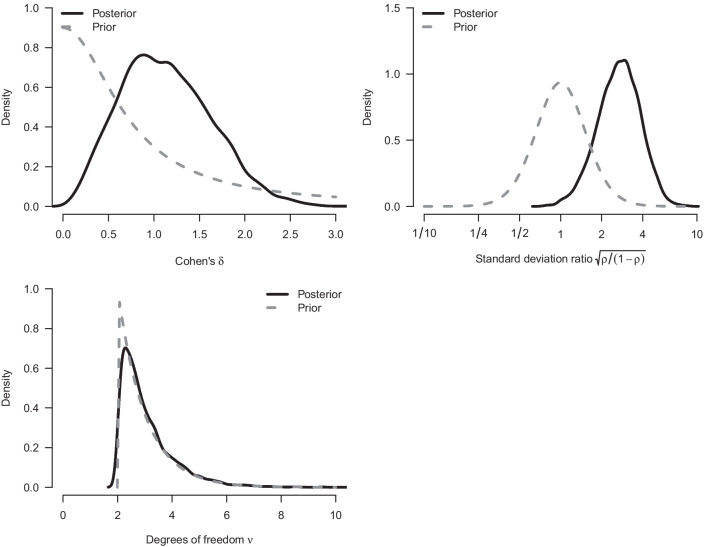
Prior posterior plot for Cohen’s $\delta$, ${\rho }$, and *t*. All panels show the conditional prior and posterior distributions assuming the parameter to be present

**Fig. 20:**
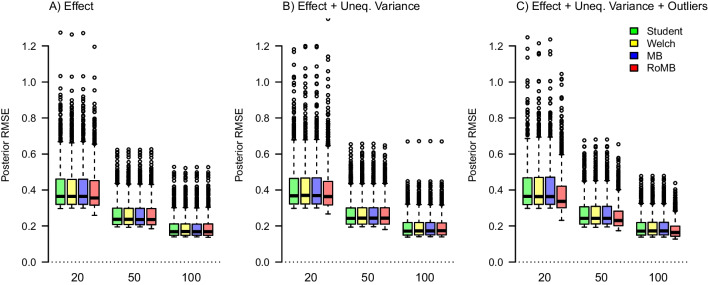
Root mean squared error (RMSE with 95% CI) of the posterior $\delta$ for different methods and conditions. The root mean squared error (and 95% CI) for the posterior distributions of the effect size $\delta$ (*y*-axis) across samples sizes (*x*-axis) for different version of the Bayesian *t* tests (in color); Student-*t* test (*green*), Welch *t* test (*yellow*), and the model-averaged version of *t* test that combines Student’s and Welch *t* test (MB; *blue*) and also incorporates uncertainty about the outliers (RoBM; *red*). Panel **A** corresponds to a condition with an effect ($\delta$ = 0.5) equal-variances and no outliers, panel **B** corresponds to a condition with an effect ($\delta$ = 0.5), unequal variances (SDR = 2), and the absence of outliers, and Panel C corresponds to a condition with the effect ($\delta$ = 0.5), unequal variances (standard deviation ratio = 2), and outliers (data simulated from a Student-*t* distribution with $t = 5$ degrees of freedom)

**Fig. 21:**
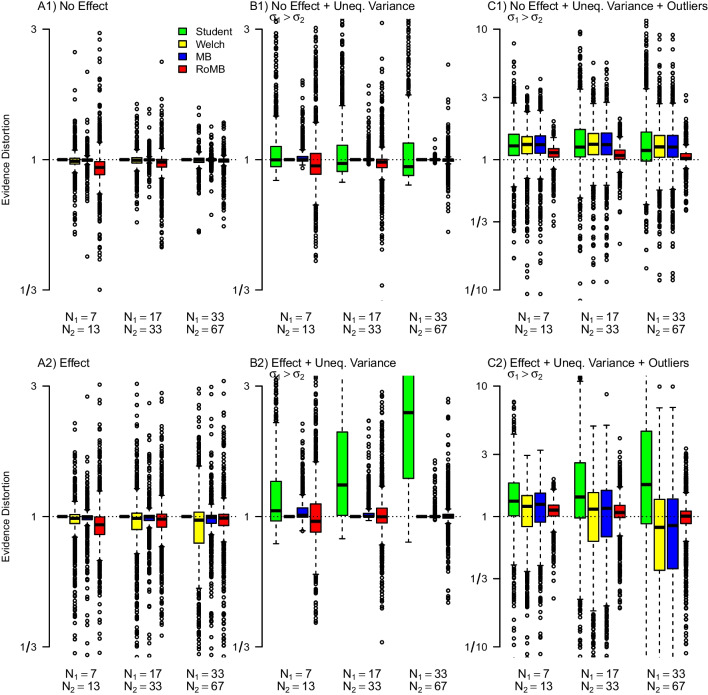
The four methods are the Student’s-*t* test (*green*), the Welch *t* test (*yellow*), a model-averaged version of *t* test that combines Student’s and Welch *t* test (MB *t* test; *blue*) and a version that also incorporates uncertainty about the outliers (RoMB *t* test; *red*). Whenever the difference in means is present (second row) then $\delta$ = 0.5. Whenever the variances are unequal (columns 2 and 3) SDR is 2, whenever the data are simulated from a *t*-distribution (column 3) this was done with $\nu$ = 5 degrees of freedom

## Data Availability

https://osf.io/mwkp6/

## Appendix A Directional perinull testing

The focus of the main text is on the comparison of a null hypothesis in which the difference in means is postulated to be exactly zero (i.e., a point null or sharp hypothesis) versus an alternative hypothesis in which the effect is assigned a continuous prior distribution. However, small deviations from the null hypothesis are often considered trivial or uninteresting (e.g., Gelman and Carlin, 2014; Good, 1967; Meehl, 1978; Orben and Lakens, 2020). Moreover, many statisticians view the point-null hypothesis as certainly false, and use it only as a mathematically convenient approximation. For these reasons it has been advocated to replace the point null hypothesis with a perinull hypothesis which assigns the difference in means a continuous distribution tightly centered around the point null value (e.g., Berger and Sellke, 1987; Cornfield, 1966; George and McCulloch, 1993). This can be done smoothly within our *t* test framework. In addition, we wish to incorporate the directional prediction of Pleasant and Barclay (2018) by using one-sided prior distribution. Consequently; we may then specify a one-sided hypothesis test against a perinull hypothesis. We specify the perinull hypothesis via a $\text {Normal}_+$(0, 0.01) prior distribution on the Cohen’s $\delta$ scale, which gives 95% credence to values within a $\pm 0.02$ interval, and compare it to the one-sided hypothesis specified by truncating the default Cauchy hypothesis at zero, $\text {Cauchy}_+$(0, 1/$\sqrt{2}$).

Comparing a directional alternative hypothesis to a perinull hypothesis, the results show strong evidence for a difference in means $\text {BF}_{+0}$ = 99.91. In addition, there still weak evidence for outliers $\text {BF}^{tn}$ = 1.18 and strong evidence for unequal variances $\text {BF}^{{\rho }\overline{\rho }}$ = 16.85.

Figure 19 visualizes the model-averaged estimate obtained by weighing the individual model estimates based on their posterior probability. Overall, we find a model-averaged mean effect size estimate $\delta$ = 1.12, 95% CI [0.24, 2.18], a model-averaged standard deviation ratio estimate SDR = 2.75, 95% CI [1.00, 5.16], and a model-averaged median degrees of freedom estimate $t = 2.79$, 95% CI [2.06 5.61].

## Appendix B Model specification

For the unequal-variance *t* test we define $\mathcal {H}_{1, 1}$ as the model with normal likelihood, unequal variances and unequal means. The normal likelihood implies that

6 $$\begin{aligned} x_{1} \sim \text {Normal}( \mu + \alpha /2, \sigma _{1}^{2}) \end{aligned}$$

7 $$\begin{aligned} x_{2} \sim \text {Normal}( \mu - \alpha /2, \sigma _{2}^{2}) \end{aligned}$$

where $\mu$ is interpreted as grant mean, $\alpha$ a difference in means as suggested by Wetzels et al. (2009). For the variances; we use the parametrization from Dablander et al. (2020). This is based on the precisions $\tau _{1} = 2 \rho \bar{\tau }$ and $\tau _{2} = 2 (1-\rho ) \bar{\tau }$ and the average precision is $\bar{\tau } = (\tau _{1} + \tau _{2}) / 2$. Defining the “common” variance by $\sigma ^{2} = 1/\bar{\tau }$ and setting $\sigma _{k}^{2} = 1/\tau _{k}$ for $k=1,2$, we then get the parametrization

8 $$\begin{aligned} \sigma _{1}^{2} = \frac{ \sigma ^{2}}{2 \rho } \quad \sigma _{2}^{2} = \frac{ \sigma ^{2}}{2 (1-\rho )} \end{aligned}$$

9 $$\begin{aligned} \alpha = \delta \sqrt{ \sigma _{1}^{2} \tfrac{n_{1}}{n_{1}+n_{2}} + \sigma _{2}^{2} \tfrac{n_{2}}{n_{1}+n_{2}}} \end{aligned}$$

The grant mean $\mu$ and common variance $\sigma ^{2}$ are nuisance, whereas the standardized effect size $\delta$ and the precision proportion $\rho$ are test-relevant parameters. For a Bayes factor we need to set priors on the free parameters. Whenever $\delta$ or $\rho$ is free to vary, we then choose

10 $$\begin{aligned} \pi (\mu , \sigma ^{2}) \propto \frac{1}{\sigma ^{2}} \end{aligned}$$

11 $$\begin{aligned} \pi (\delta ) = \text {Cauchy}(0, \frac{1}{\sqrt{2}}) \end{aligned}$$

12 $$\begin{aligned} \pi (\rho ) = \text {Beta}(1.5, 1.5) \end{aligned}$$

Note that for the unequal variances normal likelihood model $\mathcal {H}_{0,1}$, we set $\delta = 0$ and it cannot vary freely anymore. Hence, for $\mathcal {H}_{0,1}$ only the priors on $\mu , \sigma ^{2}$ and $\rho$ suffice.

### Student-*t* likelihood

We replace the normal likelihood models Eqs. 6 and 7 with

13 $$\begin{aligned} x_{1}&\sim \text {StudentT}(\mu _{t} = \mu + \alpha /2, \sigma _{t} = \frac{\sigma _{1}^{2}}{ \sqrt{\nu / ( \nu -2)}} ) \end{aligned}$$

14 $$\begin{aligned} x_{2}&\sim \text {StudentT}(\mu _{t} = \mu - \alpha /2, \sigma _{t} = \frac{\sigma _{2}^{2}}{ \sqrt{\nu / ( \nu -2)}} ) \end{aligned}$$

where $\mu _{t}$ represents the location and $\sigma _{t}$ the scale of the location-scaled t-distribution.

For the Bayes factor involving *t*-likelihood the additional free parameter $\nu$ also needs a prior distribution for which we choose

15 $$\begin{aligned} \pi (\nu ) = \text {exp}(\nu - 2) \end{aligned}$$

## Appendix C Simulation results for posterior RMSE

Figure 20 visualizes the RMSE of the posterior distribution for $\delta$ across three different conditions: (A) difference in means, with Cohen’s $\delta$ = 0.5; (B) difference in means and unequal variances with an SDR of 2; and (C) and difference in means, unequal variances, and outliers with the data sampled from a *t*-distribution with 5 degrees of freedom. All three versions of the *t* test produce comparable effect sizes estimates for the first two scenarios; differences in variances should not affect the posterior distribution since the mean difference of two normal distributions is independent of the variances of the two normal distributions. However, we see that the outliers in the third condition impact the RMSE for the posterior distribution from the Bayesian version of the Student and Welch *t* test slightly more than the model-averaged version of the *t* test. In other words, model-averaging over *t*-likelihoods comes at little cost when there are no outliers but increases accuracy when outliers are present. The reason why the RoMB models sometimes outperform the simpler models even when variances are equal and there are no outliers is related to the regularizing properties of the prior distributions. In cases, where the maximum likelihood estimate for Cohen’s *d* is larger than the true Cohen’s *d* value, the estimates will be shrunken somewhat more towards zero (i.e., the correct value). Therefore, the *t*-likelihood models can outperform the other models even when they capture the data generating process somewhat less well; however, this superiority is very small and dependent on the regularizing properties of the prior distribution.

## Footnotes

1 Sequential analyses are also possible in the frequentist framework, although this usually entails an explicit advance commitment either to the number of interim tests or the precise value of the test-relevant parameter under the alternative hypothesis (see e.g., Jennison and Turnbull, 1999; Lakens, 2014; Schnuerch and Erdfelder, 2020; Stefan et al., 2020).

2 It is sometimes advocated to use Welch *t* test by default (e.g., Delacre et al., 2017) instead of first testing for equality of variances. While this suggestion may improve the practice of testing equality of means, it does not solve the fundamental problem of basing the inference on a single model rather than considering multiple models simultaneously.

3 Cohen’s $\delta$ is the population version of Cohen’s *d*, in other words, the “true” effect size which would manifest itself in a hypothetical study with an infinitely-large sample size. Cohen’s $\delta$ is the difference in population means (rather than the sample means) scaled by the appropriate standard deviation. In an equal variance case this is $\delta$ = $\frac{\mu _{1} - \mu _{2} }{\sigma }$.

4 Note that the BayesFactor R package already incorporates solutions for this case, which are faster than our sampling-based approach (Morey & Rouder, 2015). The explanation of the equal-variance case is intended to set the stage for the extensions.

5 The order is inverted if the Bayes factor in favor of the null over the alternative is calculated.

6 The Cauchy distribution is a t-distribution with one degree of freedom; compared to the normal distribution, the Cauchy has fatter tails (see Rouder et al. (2009) for a rationale). The first parameter of the Cauchy indicates its median and the second parameter indicates its interquartile range.

7 Note that in order to fulfill additional desiderata, Dablander et al. (2020) suggest the Beta($\nicefrac {1}{2}, \nicefrac {1}{2}$) distribution as a default prior on the precision proportion. Here we use background knowledge about experimental psychology (i.e., the knowledge that very extreme standard deviations ratios are less likely than smaller ratios) and adopt an informed prior instead. The R package offers a variety of choices for specifying the prior distributions.

8 This paper was a part of the project Systematizing Confidence in Open Research and Evidence (SCORE, Alipourfard et al., 2021), a large project aiming to assess the credibility of social science findings that involves validating the preregistration of contributing studies, thus diminishing the scope for selective reporting and other biases.

9 Even after applying the *t*-distributions, there is still excess kurtosis/zero-inflation that could be modeled in future extensions incorporating a double-exponential/zero-inflated model.

10 For data sets with 100–1000 observations, it takes approximately 1–8 min to estimate all 8 models on a single processor core and running 4 Markov chains for 10,000 iterations each.

## Code Availability

https://osf.io/mwkp6/
